# Influence of kinematics and incidence angles on the cutting efficiency of two single‐file nickel‐titanium rotary instruments

**DOI:** 10.1111/aej.12543

**Published:** 2021-07-13

**Authors:** Eugenio Pedullà, Giusy Rita Maria La Rosa, Giuseppe Romano, Giuseppe Leanza, Silvia Rapisarda, Gaetano Isola, Sebastiano Ferlito, Prasanna Neelakantan, Luigi Generali

**Affiliations:** ^1^ Department of General Surgery and Medical ‐ Surgical Specialties University of Catania Catania Italy; ^2^ Discipline of Endodontology Faculty of Dentistry The University of Hong Kong The Prince Philip Dental Hospital Hong Kong SAR China; ^3^ Endodontic Section Department of Surgery, Medicine Dentistry and Morphological Sciences with Transplant Surgery, Oncology and Regenerative Medicine Relevance (CHIMOMO) School of Dentistry University of Modena and Reggio Emilia Modena Italy

**Keywords:** continuous rotation, cutting efficiency, file design, incidence angles, reciprocation

## Abstract

To compare the cutting efficiency of F6 Sky Taper (F6ST) and One Curve (OC) with different kinematics and cutting inclinations. Cutting efficiency of 80 new F6ST and OC was tested at 90° and 70° inclination in relation to the sample, in continuous rotation and reciprocation, against standardised gypsum samples for 120 seconds using a customised device. Data expressed as weight loss and length of the sample cut were analysed using two‐way analysis of variance and Tukey t‐test (P<.05). F6ST showed significantly higher cutting efficiency in reciprocation, while OC in continuous rotation. Regardless of inclination, F6ST showed statistically higher values than OC in reciprocation, while OC exhibited higher cutting ability in continuous rotation. The 70° inclination significantly improved the cutting efficiency of both files. Reciprocation improved the cutting efficiency of F6ST while continuous rotation enhanced cutting ability of OC. An inclined insertion improved the cutting ability, independently from the movement.

## Introduction

The creation of new nickel‐titanium (NiTi) endodontic instruments generated considerable clinical advantages (1); in particular, the introduction of single‐file techniques reduced the number of files needed and the possibility of producing inhomogeneous walls (2). These files include the F6 Sky Taper (F6ST, Komet, Brasseler GmbH & Co., Lemgo, Germany) and the One Curve (OC, Micro‐Mega, Besançon, France) rotary instruments (3, 4, 5, 6, 7).

One important attribute of an endodontic instrument is cutting efficiency to ensure its advancement into the root canal without the application of excessive pressing forces (2, 8). Cutting action of NiTi files is affected by several factors including file designs, angle of file access (i.e. angulated access due to contracted endoodontic access cavities known as CECs) and kinematics (9, 10, 11). With respect to the type of kinematics, the first study testing with an alternating movement was that of Yared in 2008, which employed only one ProTaper F2 instrument (Dentsply Maillefer, Ballaigues, Switzerland) in a reciprocating movement (12). Reciprocation seems to demonstrate certain advantages in terms of fracture resistance and cutting efficiency (13).

The cutting efficiency of NiTi instruments has been evaluated using different protocols (14) including the time taken by the instrument to penetrate the sample (15), the amount of material removed per expended energy unit (16) and the depth of the cut or the weight loss of the sample (1). Various authors have analysed the action of the file on the sample evaluating its axial and lateral cut applying them, respectively, inside standardised conical canals (17) or laterally on a single point of the sample (18).

To date, there are no studies comparing the influence of kinematics and incidence angle on cutting efficiency of NiTi instruments. Therefore, the purpose of the present study was to evaluate the cutting efficiency of F6ST and OC with different types of movement (continuous rotation or reciprocation) and different incidence angles against the substrate. The null hypotheses tested were as follows: there would be no difference between the different types of movement (i) and the angles tested (ii) regarding their influence on cutting efficiency; (iii) there would be no difference between the two instruments tested regarding their cutting ability.

## Materials and methods

### Instruments

Sample size calculation was based on a pilot study. Considering a test power of 0.80 (G*Power 3.1.9.2 software, Heinrich‐Heine‐Universität Düsseldorf, Düsseldorf, Germany) with α = 0.05, and the minimum sample size was established at 10 instruments for each group (n = 10). Therefore, a total of 80 rotary instruments including 40 F6ST 25.06 and 40 OC 25.06 were tested. F6ST files, made of conventional 55‐NiTi austenitic alloy, have a non‐working tip and an S‐shaped cross‐section generated by the right‐hand winding of the two cutting blades. They are designed to work in clockwise rotation (300 rpm) with a maximum torque set at 2.2‐2.8 Ncm (3, 4). OC files are composed of an intermetallic NiTi C‐Wire compound treated according to Micro‐Mega patented procedures. They use a variable cross‐section with a triangular conformation near the tip that becomes S‐shaped in the coronal part; in both portions, the winding of the blades is right‐handed. Usage parameters are continuous rotation at 300 rpm and torque set at 2.5 Ncm (5, 6, 7).

The tests were performed using a customised machine built for this purpose, equipped with a step‐by‐step motor (57HS10042A4D8; 1.8”, 4.2A, Sumtor, Nema Stepper Motor, Nair Motion Technologies Co., Ltd, JiangSu, China) managed by an Arduino hardware platform (Arduino, Smart Project Srl, Strambino, Italy) (10, 19). All new instruments were inspected using an optical stereomicroscope at ×20 magnification for morphological analysis and for any signs of deformation (13, 20).

F6ST and OC instruments were tested in continuous rotation (clockwise) at 300 rpm or in reciprocation (CW 150° ‐ CCW 30°) at the same speed at 5 mm from their tip, with a cutting inclination of 90° and 70° for each movement. Considering that reciprocation is clinically used by applying a counterclockwise (CCW) cutting action and a clockwise (CW) disengagement action due to the left‐handed arrangement of the blades, it was essential to invert the cutting angles to guarantee the use of instruments having a right‐handed blade arrangement as in the case of F6ST and OC. Torque limit was set at the maximum level of the motor used for the tests (2.5 Ncm) allowing all instruments to rotate without any interruption in continuous rotation or reciprocation.

### Samples preparation

Gypsum IV plates were used, as previously reported (Zeus Dental Stone IV, Zingardi, Italy), using standardised 3D‐printed moulds (10, 19). A dental laboratory vacuum mixer (Zhermack Labomix, Badia Polesine, Italy) was used to produce gypsum plates ensuring the same gypsum powder/ water ratio for every sample.

### Customised testing machine

**Figure 1:**
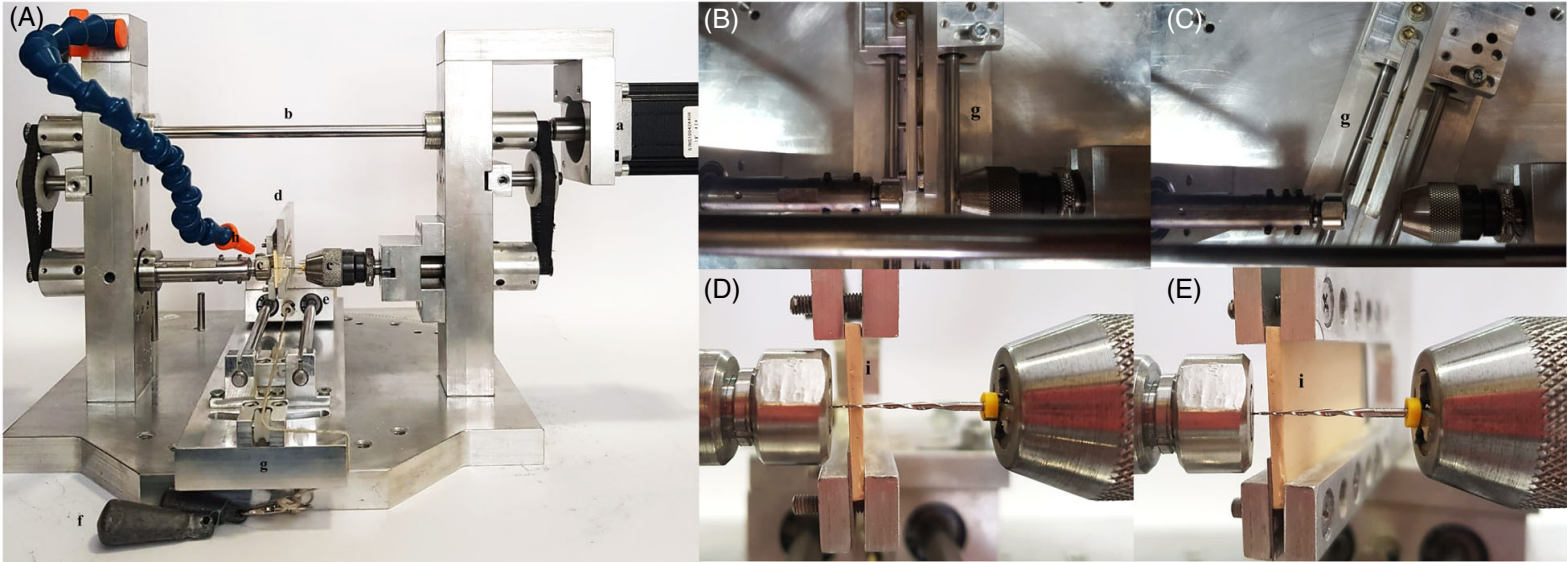
General view of customised testing machine device used for cutting efficiency tests (A) with F6Sky Taper file inserted and details of machine from above (B, C) and in frontal view (D, E) at 90° (B, D) and 70° (C, E) of inclination. A step‐by‐step motor (a) transmits the rotation by a crankshaft (b) to two chucks (c) which maintain the tested file fixed; the gypsum sample is placed on an U‐shaped support (d) connected to a mobile platform (e) and to a constant weight (f) that moves the sample against the tested instrument in an adjustable platform (g) positioned at 90° (B, D) or 70° (C, E). An air compressor (h) is used to remove gypsum debris created during the test. A new gypsum plate (i) was used for each instrument.

Cutting efficiency was tested using a customised apparatus as previously described (10, 19) (Fig. 1).

A crankshaft transmitted the rotation from the step‐by‐step motor to two chucks which maintained the tested file. Consequently, when the engine is switched on, the instrument rotated simultaneously to the two chucks.

The gypsum samples were inserted in an U‐shaped support on a mobile platform connected to a constant weight (150 g) that, because of the gravity, guided the gypsum block against the instrument always in the same standardised way, ensuring the reproducibility of the experimental conditions. Moreover, the U‐shaped support could be rotated, ensuring for different predetermined angles of incidence between the instrument and the sample.

### Cutting tests

Each instrument was tested only once. The cutting capacity was analysed at 5 ± 0.75 mm from the tip of the file for all tests.

After the setup of the testing machine and the pre‐test weighing of the sample, the file and the gypsum plate were mounted in the appropriate supports and the test started. The activated file, by cutting off the gypsum, would actively pass through it until the 120 seconds had elapsed. A compressed air jet was used to eliminate the gypsum debris created during the test and avoid clogging and, therefore, the loss of efficiency of the blades. New instruments and samples were used for each individual test (10, 19).

### Data calculation

Cutting efficiency was evaluated by the loss of mass of the sample and the length of the cut impressed by the instruments (21, 22). Each sample was subjected to a pre‐cut and post‐cut weight in order to evaluate the quantity of mass lost during the test using a precision analytical balance (± 1 × 10^‐1^mg) (ADB 200‐4 analytical balance, KERN & SOHN GmbH). The length of the sample cut was instead measured with a precision calliper (sensitivity of ± 10^‐1^ mm) (ABS Calibro Digitale AOS, Mytutoyo Italiana). The measurements for each test were performed three times taking as a reference value the average value.

### Statistical analysis

The normality of the data was verified through the Shapiro–Wilk test, and data were analysed by the two‐way ANOVA test and the post hoc Tukey test for multiple comparisons with significance level set at *P* < 0.05. The two independent variables were i) kinematics and ii) inclination angle. The principal aim of the two‐way ANOVA was to clarify whether there was an interaction between the two independent variables (i.e. kinematics and inclination angle) on the dependent variable (i.e. cutting efficiency).

## Results

The mean cutting depth and the mean weight loss of gypsum samples with standard deviations are given in Tables 1 and 2, respectively. Comparing the two evaluation methods, there were no significant differences in the results of the cutting capacities. The inferential analysis revealed significant differences between the kinematics tested, considering the angle of inclination as the independent variable (two‐way anova, *P* < 0.05; interaction *P* < 0.05); moreover, there were significant differences between the different inclination angles considering the kinematics as the independent variable (two‐way anova, *P* < 0.05).

**Table 1:** Mean ± standard deviation (SD) of cutting depth of samples at 90° and 70° instrument/sample inclinations

Instrument	*n*	Cutting depth (mm × 10^‐1^)			
		Inclination angle of the sample			
		90°		70°	
		Continuous rotation	Reciprocation	Continuous rotation	Reciprocation
		Mean ± SD	Mean ± SD	Mean ± SD	Mean ± SD
F6ST Sky Taper	10	92^a1^ ± 2	117^b1^ ± 2	123^b1^ ± 3	158^c1^ ± 3
One Curve	10	109^a2^ ± 3	86^b2^ ± 2	146^c2^ ± 2	105^a2^ ± 2

SD, standard deviation.

The same letters show differences not statistically significant (*P* > 0.05) in comparison with different groups of the same brand; the same number show differences not statistically significant (*P* > 0.05) in comparison with the same group of different brands.

**Table 2:** Mean ± standard deviation (SD) of weight loss of samples at 90° and 70° instrument/sample inclinations

Instrument	*n*	Weight loss of samples (mg × 10^‐1^)			
		Inclination angle of the sample			
		90°		70°	
		Continuous rotation	Reciprocation	Continuous rotation	Reciprocation
		Mean ± SD	Mean ± SD	Mean ± SD	Mean ± SD
F6ST Sky Taper	10	128^a1^ ± 3	159^b1^ ± 4	162^b1^ ± 4	192^c1^ ± 4
One Curve	10	147^a2^ ± 3	118^b2^ ± 3	178^c2^ ± 3	144^a2^ ± 3

SD, standard deviation.

The same letters show differences not statistically significant (*P* > 0.05) in comparison with different groups of the same brand; the same number show differences not statistically significant (*P* > 0.05) in comparison with the same group of different brands.

As for the tested movements, regardless of the inclinations (*P* > 0.05), F6ST showed a greater cutting capacity in reciprocation (*P* < 0.05), while OC obtained significantly better results in continuous rotation (*P* < 0.05).

As for the different inclinations, regardless of the tested kinematics, the 70° angle had significantly improved the cutting efficiency of both tested instruments (*P* < 0.05). Comparing the two instruments, regardless of the angle tested (*P* > 0.05), in reciprocation F6ST showed statistically higher values than OC, while in continuous rotation OC showed significantly higher values compared with F6ST (*P* < 0.05).

## Discussion

In this study, cutting efficiency was investigated using a new customised machine. This allowed greater control of the variables, ensuring a reproducible comparison of cutting properties of the tested instruments. Numerous studies discouraged to test cutting efficiency using human or bovine teeth dentine due to their variable hardness and water content (22, 23). Plexiglass has been proposed as an alternative material (22, 23); however, its hardness has been demonstrated to be lower than the dentine one (24). On the other hand, gypsum hardness [i.e. 44.91Vickers Hardness (VHN)] (25) seems to be similar with the dentine one (i.e. 46‐53 VHN) (26). For this reason, gypsum was chosen because of hardness comparable with the one of root canal dentine and its homogeneous composition due to the carefully standardised production as in the methodology described (10, 19).

Each instrument was used only once to avoid an efficiency loss due to multiple uses of the same file (10, 22). The customised machine, through its components designed to keep the instrument in axis, allowed to test instruments up to 5 ± 0.75 mm from the tip without the deflection caused by the impact of the sample (10, 19). We tested cutting efficiency of files at 5mm from their tip area because it is one of the instrument portion surely involved during the clinical procedures and engaged in a part of root canal clinically relevant (19).

Both the type of movement and the angles greatly affect the performance of the instruments. The complexity of anatomical curvatures and angulated file access induced by contracted endodontic cavities (CECs) cause an inhomogeneous contact between the file blades and the dentinal walls thus affecting the cutting efficacy (10, 11). Alovisi *et al*. (11) reported the mean angle of file access in a mesial canal of a first mandibular molar was approximately 20° with a traditional endodontic cavity and 20°–30° with a contracted endodontic access cavity. Thus, the angles chosen are clinically relevant because they offer a reliable simulation of conditions in which an instrument can be more inclined (i.e. 70°) such as in the contracted/ninja endodontic cavities or with a straighter access such as in the traditional endodontic cavity (i.e. 90°) (10, 11)

In addition, cutting efficiency is also dependent on instrument motion (22). F6ST and OC files were chosen because are both single files, available in 25.06 size, of common use among clinicians. In addition, to the best of our knowledge, no studies have evaluated the effects of kinematics and incidence angles on their cutting ability. In this context, it is important to emphasise that F6ST and the OC are designed exclusively for use in continuous rotation since their right‐handed conformation would prevent the cutting action in the classical reciprocation mode. Thus, in the current study, it was essential to invert the cutting angles in reciprocation to guarantee the use of instruments having a right‐handed blade arrangement as in the case of F6ST and OC (27). The use of reciprocation in this experimentation with inversion of the cutting and release angle had the purpose of highlighting the possible advantages or disadvantages produced by different designs combined with different movements.

Kinematics and file inclination were analysed in order to evaluate their interaction on cutting efficiency and verify as two common clinical variables could act synergistically on cutting efficiency behaviour. Moreover, no data are available in the literature on the combined effect of kinematics and file inclination on cutting action of NiTi files. In addition, endodontic files can be used in two fundamental actions: laterally or in a so‐called ‘brushing’ motion and axially (28). In the present study, lateral cutting action of the F6ST and OC was evaluated in a standardised manner which ensured the reproducibility of the methods described.

In this study, both the tested instruments exhibited a significant different cutting behaviour in continuous rotation and reciprocation. Therefore, the first null hypothesis was rejected.

As for the F6ST, comparing the two movements, the reciprocation showed a significant increase in cutting efficiency at both inclinations of 90° and 70°. The greater cutting capacity obtained by reciprocation is probably due to the S‐shaped design of blades of tested instrument (3, 4). Previous studies reported how S‐shaped cross‐section, created by the arrangement of only two cutting edges and a positive rake angle, are associated with a better cutting efficiency in continuous rotation and in reciprocation (9, 17, 22, 29, 30). Moreover, advantages of reciprocation could be due to the intrinsic characteristics of movement that seems to maintain greater cleaning of the blades. Previous studies on Reciproc files showed that reciprocation (150° CCW and 30° CW) was fundamental in the flow of debris, and therefore in the cutting efficiency increase (9, 22). A constant air compressor‐blowing has optimised this dynamic by sweeping away the debris produced (22).

Regarding cutting behaviour of OC, comparing the two movements, the use of continuous rotation showed a greater increase in cutting efficiency at both inclinations of 90° and 70°. The greatest cutting efficiency shown in continuous rotation would seem to depend on the triangular section created by the presence of three blades in the most apical section as shown by scanning electron microscope (SEM) investigations of previous studies (5, 31, 32, 33). In this study, the instruments were tested at a distance of 5 ± 0.75 mm from the tip, and consequently, it is conceivable the working portion was the one with the triangular section. On the basis of these results, it is possible to hypothesise that the three blades improved the removal of the material by increasing the extension of the instrument / sample contact surfaces exploiting advantage of the complete rotation at 300rpm and thus creating a greater number of contacts for each complete rotation of 360°. On the other hand, during reciprocation, the release angle combined with the three cutting edges can reduce the mechanical removal of debris, blocking them between at least two blades and the sample, thus generating a progressive accumulation and negatively affecting the activity of the blades. Furthermore, it should be considered that the reduction in the flutes width due to the presence of three blades and their proximity would allow a further reduction in the blade/sample contact (2). Therefore, these files benefit from continuous rotation, but not from reciprocation. In accordance with the studies conducted by Gambarini (34) and Plotino (9), reciprocation advantaged the files with two blades (i.e. Reciproc) compared with those with three blades (i.e. WaveOne) (9, 34). Nevertheless, the considerations regarding the OC design should be further investigated and additional studies are necessary to confirm these preliminary hypotheses.

Regarding the different incidence angles, an inclination of 70° produced in the two tested files an increase in their cutting efficiency independently from the type of movement tested. Consequently, also the second null hypothesis was rejected. As previously reported (10), the better cutting efficiency observed at 70° is probably due to the greater contact between the instrument area and sample. Indeed, the more instrument is inclined, and the more active blades can work at the same time on gypsum sample removing more debris.

Comparing the two instruments and using the same parameters, the OC exhibited higher cutting efficiency than the F6ST in the continuous rotation, while in reciprocating motion, the F6ST had higher values. These results are likely due to the benefits provided by the two types of cross‐sectional designs (i.e. the S‐shaped for F6ST and the triangular for OC at 5 mm) in different kinematics patterns, as previously described (5, 22, 29, 32). Also, the third null hypothesis was rejected. As already stated, contemporary literature does not present studies on the cutting capacity of the tested instruments. In particular, since there are no direct comparisons of the cutting efficiency of F6ST and OC files, direct comparisons of this data are not possible.

These results must be extrapolated to practice with prudence due to the different clinical variables implicated that are difficult to reproduce simultaneously in clinical practice (35). These variables include anatomy (i.e. teeth inclination, root canal configuration and dentin composition), type of instrument (geometrical features and surface treatment), environmental temperature and dynamical process of an endodontic treatment. In addition, each instrument in clinical performance may have different deformation due to the elastic and/or plastic deformations caused by applied stress that also influences cutting efficiency. Another potential limitation to consider is that the tests were conducted in static mode without simulation of pecking motion. This approach was necessary to standardise the procedure and making reproducible the tests performed and give also some useful indications on how the files work laterally. Nevertheless, the brushing action does not refer only to lateral cutting but also to pecking motion whose amplitude can continuously change in clinical practice and cannot be standardised in predictable manner. Thus, the results obtained should be clinically evaluated with caution. Further studies are needed to confirm these in vitro results.

As concerns for clinical implications, the results obtained offer useful indications to the clinician on how cross‐sectional design combined with file kinematics can affect cutting efficacy of NiTi files. With regard to cutting action, when a clinician chooses an instrument, should be aware as a S‐shaped cross‐section is likely advantaged by a reciprocating motion while a three‐blades design could be more performant in continuous rotation. Furthermore, on the basis of our results, because the insertion into canal is rarely straight, the clinician should know the possible influence of file inclination on cutting efficiency of NiTi files. In particular, the operator should be conscious that efficient cutting action of NiTi files enhances their advancement into the root canal, reducing their possible blockage and torsional fracture (8).

The cutting efficiency of endodontic instruments depends on multiple factors including the sample/instrument inclination, file design and the kinematic used. Specifically, reciprocation brings advantages for instruments with S‐shaped cross‐section such as F6ST, while continuous rotation seems to favour instruments with a triangular section such as OC. In addition, a more inclined contact between the sample/instrument improves the cutting abilities of F6ST and OC, independently of the tested motion.

## Disclosure statement

No financial support was received. All authors deny any conflict of interest.

## Authorship

All authors have contributed significantly, and all authors are in agreement with the manuscript.
